# Identification of a Nuclear Respiratory Factor 1 Recognition Motif in the Apolipoprotein E Variant APOE4 linked to Alzheimer’s Disease

**DOI:** 10.1038/srep40668

**Published:** 2017-01-17

**Authors:** Anne Urfer-Buchwalder, Roman Urfer

**Affiliations:** 1 Selonterra LLC, 1025 Alameda de las Pulgas, Suite 126, Belmont CA 94002, USA

## Abstract

Alzheimer’s disease affects tens of millions of people worldwide and its prevalence continues to rise. It is caused by a combination of a subject’s heredity, environment, lifestyle, and medical condition. The most significant genetic risk factor for late onset Alzheimer’s disease is a variant of the apolipoprotein E gene, APOE4. Here we show that the single nucleotide polymorphism rs429358 that defines APOE4 is located in a short sequence motif repeated several times within exon 4 of apolipoprotein E, reminiscent of the structure of transcriptional enhancers. A JASPAR database search predicts that the T to C transition in rs429358 generates a binding motif for nuclear respiratory factor NRF1. This site appears to be part of a binding site cluster for this transcription factor on exon 4 of APOE. This *de novo* NRF1 binding site has therefore the potential to affect the expression of multiple genes in its genomic vicinity. Our *in silico* analysis, suggesting a novel function for APOE4 at the DNA level, offers a potential mechanism for the observed tissue specific neurodegeneration and the role of environmental factors in Alzheimer’s disease etiology.

Human apolipoprotein E (APOE) plays a key role in the regulation of lipid transport in the central nervous system and in the plasma through its interaction with low-density lipoprotein receptors^1^ and it is involved in many other biological processes not directly linked to its lipid transport function^2^. The APOE gene is polymorphic arising from different alleles - designated ε 2, 3 and 4 - at a single gene locus^3^. The three major isoforms, APOE *ε2* (APOE2), APOE *ε3* (APOE3), and APOE *ε4* (APOE4), differ from one another by single nucleotide C/T transitions at two locations in exon 4 of APOE, resulting in a cysteine/arginine substitution at two positions affecting residues 130 and 176 in the synthesized protein containing the signal-peptide and residues 112 and 158 in the mature APOE protein^4^. APOE3 evolved from the ancestral allele APOE4^5^ and represents the allele with the highest frequency in the human population of the present time. It is thus considered the normal isoform for APOE functions^2^. APOE2 is associated with the genetic disorder type III hyperlipoproteinemia^3^. The APOE4 allele was linked to Alzheimer’s disease in late-onset familial and sporadic Alzheimer’s disease^6^,^7^,^8^ and genome wide association studies^9^ confirmed the APOE4 locus as the most significant genetic risk factor for Alzheimer’s disease. The risk of developing Alzheimer’s disease increases with each copy of the APOE4 variant compared with the APOE3*/*APOE3 genotype: The odds ratio (OR) is 2.6 (APOE2*/*APOE4) and 3.2 (APOE3*/*APOE4) with one copy of the APOE4 allele, and the OR increases to 14.9 with two copies of the allele (APOE4*/*APOE4)^10^. On the other hand, the APOE2 allele of APOE is protective against Alzheimer’s Disease, with an OR = 0.6 for APOE2*/*APOE2 individuals. APOE4 is associated with an earlier age of onset with age 68 as mean age of clinical onset for APOE4 homozygotes versus 84 years of mean age of clinical onset for subjects not carrying the APOE4 allele^8^. Clinical and epidemiological data have indicated that, depending on the population and the study, between 40 to 80% of Alzheimer’s disease patients are APOE4 carriers^11^ with penetrance of homozygous APOE4 estimated to be at 60–80%^12^. These data show that the magnitude of the effect of APOE4 on Alzheimer’s disease is more similar to the one observed for major genes in Mendelian diseases such as BRCA1 in breast cancer than to low-risk common alleles identified by recent genome-wide association studies in complex diseases^13^. A series of hypotheses have been proposed to explain the association of the APOE4 allele with Alzheimer’s disease: impairment of the antioxidative defense system, dysregulation of neuronal signaling pathways, disruption of cytoskeletal structure and function, altered phosphorylation of microtubule associated protein tau (MAPT) and the formation of neurofibrillary tangles, depletion of cytosolic androgen receptor levels in the brain, potentiation of Aβ-induced lysosomal leakage and apoptosis in neuronal cells, or promotion of endosomal abnormalities linked to Aβ overproduction (reviewed in ref. 14). In the brain, apolipoprotein E is expressed by about 75% of astrocytes under normal conditions with the highest level of expression in the olfactory bulb and Bergmann glia in the cerebellum^15^,^16^. Neuronal expression in human brain tissue is barely detectable but is increased in areas affected by ischemia^17^. Several Apoe mouse models have been established to study the mechanisms underlying the pathogenic actions of APOE4 and its potential relationship to Alzheimer’s disease pathology. However, expression of APOE4 in astrocytes under the control of the glial fibrillary acidic protein promoter did not lead to typical Alzheimer’s like neuropathology^18^ nor did aged APOE4 transgenic mouse brains demonstrate any evidence of senile plaques^19^. Further, APOE isoforms were expressed under the control of the physiological mouse promoter in Apoe*−/−* mice to investigate their roles on cardiovascular function^20^. Mice having targeted replacements of the intrinsic murine Apoe gene with the three human APOE alleles recapitulate many of the phenotypic cardiovascular effects seen in humans with these same isoforms^20^. Even though APOE4 stimulated the accumulation of Aβ42 and hyperphosphorylated tau in these animals at 4 months of age, the formation of tangles and senile plaques was not reported^21^. Therefore, the effects of APOE protein isoforms on cholesterol and lipid metabolism are faithfully represented in animal models but these models do not display typical Alzheimer’s disease hallmarks as a consequence of human APOE4 isoform protein expression. Further, it is striking that most mammals carry the APOE4 isoform at position 130 (Arg) indicating that this protein structure is sufficient to perform all physiological functions of APOE. Given these considerations, we hypothesized that the APOE4 allele may not cause Alzheimer’s disease solely due to its resulting change in the protein sequence but may act at the DNA level to control the expression of genes located in the vicinity of APOE4. To this end, using a bioinformatics approach, we examined the APOE exon 4 for the presence of sequence elements typically observed in transcriptional enhancers, including transcription factor binding motifs and short repeat sequences.

## Results

### Analysis of the DNA Sequence Overlapping the APOE ε Alleles

**Figure 1:**
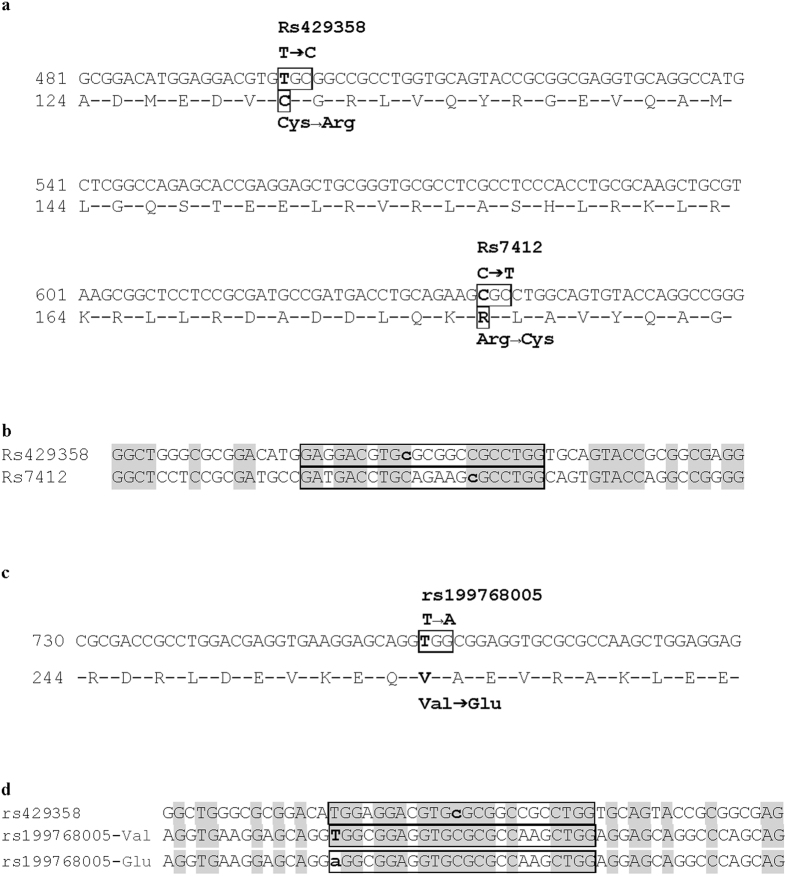
Definition of the APOE4 motif. (**a**) Location of the APOE *ε* variants on APOE exon 4. The allelic nucleotides are in bold inside the box delineating the variable codons. The resulting amino acid changes are in bold and boxed. *APOE* cDNA sequence and non-processed protein amino acid numbering are according to Ensembl transcript ENST00000252486. (**b**) Comparison of the DNA sequence encompassing the SNPs determining the APOE *ε* alleles. This alignment shows the APOE4 allele (C at position 19:44908684 and position 19:44908822 in GRCh38.p2 assembly, indicated in bold lowercase). Identical nucleotides are shaded in grey. The sequence motif with highest similarity encompassing both SNPs is boxed. (**c**) APOE*2/V236E variant. The allelic nucleotide is in bold inside the box delineating the variable codon. The resulting amino acid change is shown in bold and boxed. V236 is identified as V254 in Ensembl transcript ENST00000252486. (**d**) Sequence comparison encompassing rs429358/APOE4 and rs199768005/V236E. Common nucleotides are shaded in grey. The sequence motif with highest similarity encompassing all three SNPs (rs429358, rs7412, rs199768005) is boxed.

The APOE *ε* alleles are determined by two SNPS, rs429358 and rs7412. APOE4 harbors a C at position 19:44908684 and position 19:44908822. APOE2 harbors a T at both positions, while APOE3 harbors a T at position 19:44908684 and a C at position 19:44908822. Both SNPs are located inside exon 4 of the APOE gene, 138 bp apart (Fig. 1a). We aligned the DNA genomic sequences overlapping these SNPs and observed that they display a high degree of similarity. A core segment including the two SNPs and their immediate vicinity displayed a 68% identity with no gaps in a stretch of 22 nucleotides (Fig. 1b). APOE*2 is a rare variant situated in the lipid binding region of APOE, in which valine 236 is substituted by glutamic acid (V254E in the full length sequence, rs199768005, Fig. 1c). This variant is significantly associated with a marked reduction in risk of Alzheimer’s disease (P = 7.5 × 10−5; OR = 0.10 [0.03 to 0.45])^22^. We noticed that the DNA sequence harboring this SNP is also very similar (75% with no gaps in a segment of 24 nucleotides) to the DNA sequence encompassing rs429358, the genetic variant determining the APOE4 status (Fig. 1d). The DNA sequence encompassing rs7412 displays a similarity of 63% to this 24 nucleotide long motif. These observations of a high degree of DNA sequence similarities in three separate regions (i.e., rs429358, rs7412, rs199768005) affecting the susceptibility to Alzheimer’s disease led us to define the sequence “TGGAGGACGTG**C**GCGGCCGCCTGG” as the “APOE4 motif” (the rs429358 nucleotide observed in APOE4 is highlighted with bold/underline).

### Analysis of APOE Exon 4 DNA Sequence

**Figure 2:**
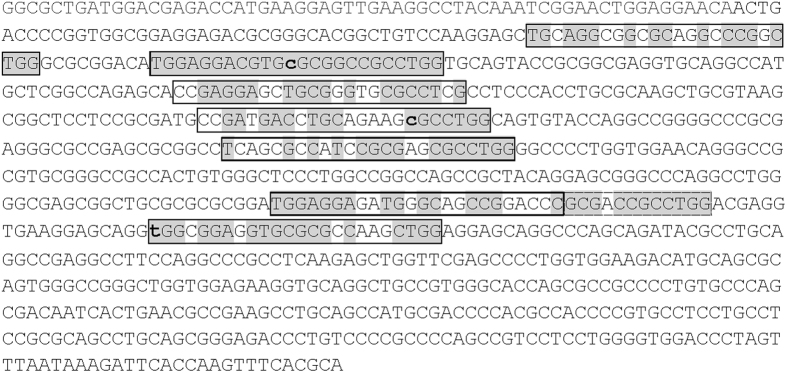
Sequence repeats within exon 4 of apolipoprotein E. Sequence shown starts with the first nucleotide of exon 4 (ENSE00000893954). Rs429358, rs7412 and rs199768005 are indicated by bold lowercase letter. For rs429358 the APOE4 nucleotide (C) is shown. For rs7412 the APOE3 nucleotide is shown (C). For rs199768005 the Val variant (T) is shown. Elements of high similarity with APOE4 motif (≥63% matches within 24 nucleotide motif with no gaps) are indicated by boxes, common nucleotides are shaded in grey. The dashed line box indicates the 5′ end of two overlapping APOE4 motifs.

Variants *ε2, ε3, and ε4* are imbedded in a CpG island (CGI) overlapping the end of intron 3 and exon 4 of the APOE gene that is highly methylated in the human brain. This APOE CGI can function both as a transcriptional enhancer or silencer in a luciferase-based reporter system depending on cell type and promoter construct^23^,^24^. Enhancers generally represent a modular arrangement of short sequence motifs, each interacting with a specific cellular transcription factor or regulatory protein, which will be responsible for turning the transcription on or off in a different set of cells, or at different times^25^. Given the observed activity of the APOE exon 4 on gene transcription^23^,^24^ and our identification of the APOE4 motif within Alzheimer’s disease determining SNPs, we inspected the exon 4 DNA sequence for the presence of additional APOE4 motif-like structural elements. This search revealed the presence of the 24 nucleotide-long APOE4 motif in 8 locations on APOE exon 4 with at least the same level of identity to the consensus with no gaps (Fig. 2) as observed within the three sequence elements defined by the three AD-associated SNPs (63%). The 8 occurrences in 5′ to 3′ order were 67%, 100%, 67%, 63%, 63%, 63%, 79% and 75% identical to the APOE4 motif (Fig. 2). Hence it appears that exon 4 of APOE harbors a modular short-sequence arrangement typical of enhancers. These repeats however were found nowhere else in the APOE gene.

### Prediction of a NRF1 Transcription Factor Binding Site within the APOE4 Motif Sequence

**Figure 3:**
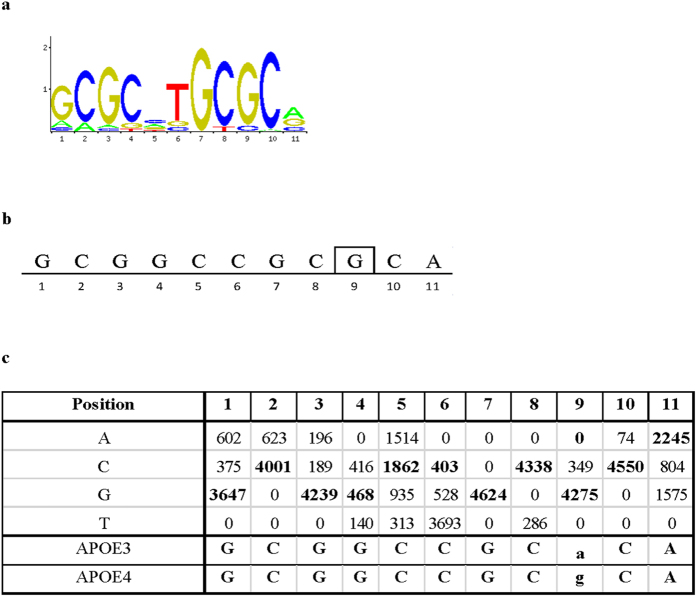
APOE4 creates a *de novo* NRF1 binding motif. (**a**) Sequence logo representing the consensus NRF1 binding motif (JASPAR database). (**b**) *De novo* NRF1 binding motif overlapping APOE4 on the reverse strand. Variant rs429358 is indicated by a box. (**c**) Position frequency matrix for each nucleotide in the NRF1 binding motif matched to APOE4 and APOE3 overlapping sequences. Variant rs429358 is indicated in small cap (reverse strand). Frequency values in APOE3 and APOE4 are in bold. Note values at position 9 for APOE3 (“A”, 0) and APOE4 (“G”, 4275).

Most enhancers exert their regulatory function through binding of cell-type specific transcription factors. Thus, we performed an *in silico* search of the DNA sequence of the APOE4 motif for putative transcription factor binding sites using binding profiles from the JASPAR CORE database of experimentally defined transcription factor binding sites for eukaryotes. A score is calculated for the probed sequence that provides a measure of similarity to the transcription factor consensus sequence. We submitted the APOE3 DNA sequence to the same query for comparison purposes. Results of the analysis are presented in Table 1, and show that the region of interest (APOE4 motif) leads to statistically significant hits for two transcription factor binding motifs, HIF1A::ARNT (Hypoxia-inducible factor 1, alpha::Aryl hydrocarbon receptor nuclear translocator) and NRF1 (Nuclear Respiratory factor 1). HIF1A::ARNT is a heterodimeric transcription factor composed of the alpha subunit HIF1A, and the beta subunit ARNT. A binding motif for this transcription factor was found in both the APOE4 and the APOE3 sequence, with similar scores of 11.2 and 9.6, respectively. The T to C transition is situated at the edge of the consensus motif in the predicted binding site sequence, a position where every nucleotide can be found with similar frequency. The nucleotide change in the APOE3 to APOE4 transition is thus not expected to affect binding. More importantly, screening of the APOE4 motif identified a binding motif for NRF1 with a score of 11.9. The NRF1 binding motif was not identified in the APOE3 query sequence when a stringent relative profile score threshold cut-off of 90% (Table 1) was applied. Hence, the rs429358 T to C transition in the APOE4 motif creates a novel consensus binding motif for NRF1 (Fig. 3a). This predicted binding site is located on the reverse strand (Fig. 3b) which is not unusual as enhancer sequences can be positioned in both forward or reverse orientations, inside, downstream, or upstream of the regulated gene and most transcription factor binding sites can occur in both orientations in promoters or enhancers. In order to assess the relative strength of the NRF1 binding motif in APOE4, a second JASPAR screen with a lower relative profile score threshold cut-off of 80% was performed (Table 2). Under these less stringent conditions, the novel NRF1 binding motif in APOE4 retained its score of 11.9 (Table 1) while the APOE3 sequence resulted in a score of 3.9 (Table 2). As a comparison, the highest score to be expected for the NRF1 consensus sequence in JASPAR is 18.1 while a score of 0 signifies that the sequence has equal probability of being a functional or a random site. Moreover, the APOE4 variant changes a non-consensus T nucleotide (A on the reverse strand) present in APOE3 with 0 appearance in the nucleotide frequency matrix of the NRF1 consensus sequence into a highly conserved, consensus matching C nucleotide (G on the reverse strand) with 4275 appearances in the nucleotide frequency matrix (Fig. 3c).

### Presence of Additional NRF1 Binding Motifs in APOE Exon 4

**Figure 4:**
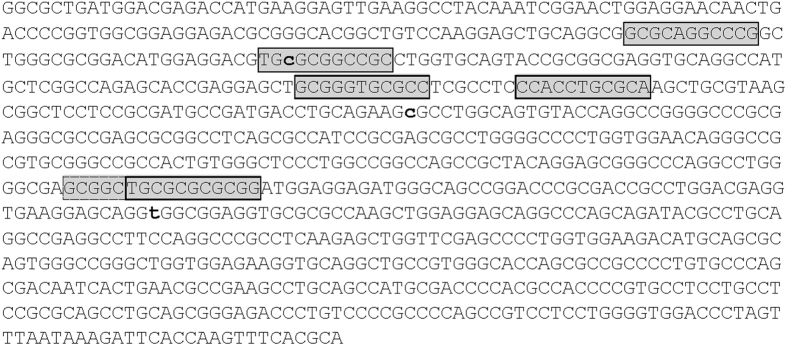
NRF1 binding motif positions on exon 4 of apolipoprotein E. Sequence shown starts with the first nucleotide of exon 4 (ENSE00000893954). Positions of predicted NRF1 binding sites are shown in boxes shaded in grey. Note that the last two sites overlap in forward and reverse orientations. Rs429358, rs7412 and rs199768005 are shown in bold small caps. For rs429358 the APOE4 nucleotide (C) is shown. For rs7412 the APOE3 nucleotide is shown (C). For rs199768005 the Val variant (T) is shown.

Clustering of multiple transcription factor binding sites for the same transcription factor – so called homotypic clusters of transcription factor binding sites are a prevalent feature of human cis-regulatory elements. These transcription factor clusters can be found both in distant enhancer elements and in promoter regions, and appear to play an active role in gene regulation^26^. Thus, we investigated whether other NRF1 binding motifs could be detected on APOE exon 4. We subjected the entire sequence of APOE exon 4 to a JASPAR database search of NRF1 binding motifs. Six NRF1 binding sites with scores ranging from 10.5 to 14.5 were predicted when a stringent relative profile score threshold of 90% was applied (Table 3). Locations of these NRF1 binding motifs on the exon 4 sequence are shown in Fig. 4. As a comparison, screening of the neighboring APOE intron 3 did not lead to any hits for NRF1.

## Discussion

We have shown in this study using a bioinformatics approach that the DNA sequences spanning polymorphisms linked to Alzheimer’s disease are conserved, and contain short sequence spans of what we defined as the APOE4 motif. We have shown that this DNA motif is repeated several times within exon 4 of apolipoprotein E, which harbors these Alzheimer’s disease alleles. Moreover, our *in silico* analysis of transcription factor binding sites using the JASPAR 2014 database revealed that the change of the T nucleotide (APOE3) to a C nucleotide (APOE4) is sufficient to create a *de novo* NRF1 binding motif. We suggest that the peculiar structural feature on exon 4 could function as a transcriptional enhancer element and be implicated in the machinery that regulates DNA transcription in the genomic vicinity of APOE4. Transcriptional enhancer elements can control transcriptional activity of genes located on the same (cis) chromosome or on different (trans) chromosomes. In the case of cis transcriptional activation, 98% of chromatin loops anchored at a promoter are located within a range of 2 Mb of the enhancer’s location^27^, indicating that the vast majority of genes regulated by the enhancer are located within 2 Mb of the enhancer’s chromosomal position. Hence, the *de novo* APOE4 NRF1 binding site could regulate multiple genes on chromosome 19 located within this genomic distance of the APOE gene. Our finding of a single nucleotide change leading to the generation of a *de novo* NRF1 site in APOE4 is in line with other studies that have shown that single nucleotide variants can affect gene expression. For example, the blond-associated allele at rs12821256 alters a binding site for the lymphoid enhancer-binding factor 1 (LEF1) and reduces LEF1 transcription factor responsiveness in keratinocytes^28^. Preaxial polydactyly, a frequently observed congenital limb malformation, results from single point mutations within the Sonic Hedgehog (SHH) regulator, designated ZRS, which lies within intron 5 of the LMBR1 gene 1 Mb from its target gene^29^,^30^. The importance of disease-associated allele polymorphisms affecting transcription has recently been highlighted in neurodegenerative disorders. Notably, it was demonstrated that a polymorphic NRF2/sMAF binding site in MAPT (Tau) is strongly associated with differential risk for Parkinson’s disease^31^. Further, a risk variant for Parkinson’s disease in a distal enhancer of alpha synuclein (*SNCA*) was shown to modulate target gene expression^32^. NRF1 is a homodimeric transcription factor that mediates the expression of key metabolic genes and of a range of nuclear genes essential for mitochondrial biogenesis^33^, including subunits of the respiratory chain complexes, and constituents of the mtDNA transcription and replication machinery. NRF1 plays an important role in the coupling between energy consumption, energy generation, and neuronal activity^34^. NRF1 has also been associated with the regulation of neurite outgrowth^35^, glucose metabolism^36^, response to exogenous oxidants^37^ and hepatitis B infection^38^. Moreover, the expression of NRF1 is increased in aged subjects^39^. NRF1 has also been found to be a potentially important factor for Alzheimer’s disease using network topology analysis of microarray data from post-mortem brains^40^. In addition, a panel of neurodegenerative disease-related genes, such as PARK2, PINK1, PARK7, GPR37, PSENEN, and MAPT have been recognized as NRF1 targets^41^. Traumatic brain injury, episodes of brain ischemia, poorly controlled diabetes as well as common infections are known risk factors influencing Alzheimer’s disease onset, progression and outcome, apart from advanced age. Thus, the NRF1 binding motif created by the APOE4 variant offers a potential mechanism to link these environmental signals to aberrant gene expression causing Alzheimer’s disease. The preferential expression of the APOE protein in glia of the cerebellum and olfactory bulb is difficult to reconcile with the well documented histopathological progression of AD^42^. Gene expression mediated by the NRF1 binding motif could provide for a mechanism for the observed tissue specificity of AD neurodegeneration. The functional role of the predicted NRF1 recognition motif on the expression of genes within the genomic vicinity of APOE and how these genes link to AD neurodegeneration will be elucidated by biochemical and molecular studies.

## Methods

### Single Nucleotide Polymorphisms and Reference Sequences

Apolipoprotein E, or APOE, HUGO Gene Nomenclature Committee ID, HGNC:613, Ensembl: ENSG00000130203, UniProtKB: P02649. APOE cDNA sequence and amino acid numbering are according to Ensembl transcript ENST00000252486. Single Nucleotide Polymorphism (SNP) rs429358 is a T/C variation that occurs inside the coding sequence of the APOE gene at position 44908684 on chromosome 19 in the Genome Reference Consortium Human Build 38 patch release 2/GRCh38.p2. SNP rs7412 is a C/T variation occurring at position 44908822 on chromosome 19 in GRCh38.p2. rs199768005 is a T/A polymorphism situated at position 19:44909057 in GRCh38.p2, resulting in the missense mutation of a valine residue into a glutamic acid (V254E in transcript ENST00000252486 or V236E in the mature protein).

Nuclear respiratory factor 1, or NRF1, is also known as ALPHA-PAL, HGNC: 7996, Ensembl:ENSG00000106459, UniProtKB: Q16656.

### Search for sequence similarities within APOE gene

Search for sequences similar to the APOE4 motif within the APOE gene was performed using NCBI Homo sapiens Nucleotide Basic Local Alignment Search Tool/Blastn (http://blast.ncbi.nlm.nih.gov/) using the default parameter values for short sequences.

### Prediction of Transcription Factor Binding Sites created by APOE4

Transcription factor binding sites were predicted by the software JASPAR 2014^43^ (http://jaspar.genereg.net/). JASPAR is an open-access collection of curated, non-redundant set of profiles, derived from published collections of experimentally defined transcription factor binding sites for eukaryotes. Sensitivity and specificity are affected by the relative score threshold (default 80%). Submitted sequences were analyzed using a relative profile score threshold setting of 90% to the “CORE Vertebrata” database, to report only the most likely sites^44^ as experimentally reported binding sites in DNA frequently locate true sites as the highest-scoring sequences^45^. Position Frequency Matrix cell numbers indicate the number of sequences having base x in column y. Sequence logos^46^ are graphical representation of a transcription factor consensus binding site, in which nucleotides are sized and sorted relative to their occurrence at each position. Ranges are from 0 (no base preference) to 2 (single base occurrence).

## Additional Information

**How to cite this article**: Urfer-Buchwalder, A. and Urfer, R. Identification of a Nuclear Respiratory Factor 1 Recognition Motif in the Apolipoprotein E Variant APOE4 linked to Alzheimer’s Disease. *Sci. Rep.* **7**, 40668; doi: 10.1038/srep40668 (2017).

**Publisher's note:** Springer Nature remains neutral with regard to jurisdictional claims in published maps and institutional affiliations.

## Figures and Tables

**Table 1:** JASPAR analysis of the region overlapping rs429358.

Model ID	Model name	Score	Start	End	Strand	Predicted site sequence
APOE4						
MA0259.1	HIF1A::ARNT	11.2	5	12	Forward	GGACGTGc
MA0506.1	NRF1	11.9	10	20	Reverse	GCGGCCGCgCA
APOE3						
MA0259.1	HIF1A::ARNT	9.6	5	12	Forward	GGACGTGt

APOE4 queried sequence was TGGAGGACGTGcGCGGCCGCCTGG. APOE3 queried sequence was TGGAGGACGTGtGCGGCCGCCTGG. Small caps indicate the APOE3/4 rs429358 polymorphism.

**Table 2:** NRF1 recognition site in APOE4 motif.

Model ID	Model name	Sequence name	Score	Strand	Predicted site
MA0506.1	NRF1	Consensus	18.1	Forward	GCGCCTGCGCA
MA0506.1	NRF1	APOE4, Rs429358/C	11.9	Reverse	GCGGCCGCgCA
MA0506.1	NRF1	APOE3, Rs429358/T	3.9	Reverse	GCGGCCGCaCA

APOE variant rs429358 is indicated in small cap.

**Table 3:** Predicted NRF1 binding sites on APOE4 exon 4.

Model ID	Model name	Score	Start	End	Strand	Predicted site sequence
MA0506.1	NRF1	10.9	116	126	Forward	GCGCAGGCCCG
*MA0506.1*	NRF1	*11.9*	*150*	*160*	*Reverse*	*GCGGCCGCgCA*
MA0506.1	NRF1	12.6	217	227	Forward	GCGGGTGCGCC
MA0506.1	NRF1	10.5	235	245	Forward	CCACCTGCGCA
MA0506.1	NRF1	14.5	454	464	Forward	GCGGCTGCGCG
MA0506.1	NRF1	10.7	459	469	Reverse	CCGCGCGCGCA

*De novo* NRF1 binding motif present in the APOE4 variant is indicated in italic.

## References

[b1] Eichner J. E., Dunn S. T., Perveen G., Thompson D. M., Stewart K. E. & Stroehla B. C. Apolipoprotein E polymorphism and cardiovascular disease: A HuGE review. Am J Epidemiol 155, 487–495 (2002). 11882522 10.1093/aje/155.6.487

[b2] Mahley R. W. & Rall S. C. Jr. Apolipoprotein E: far more than a lipid transport protein. Annu Rev Genomics Hum Genet 1, 507–37 (2000). 11701639 10.1146/annurev.genom.1.1.507

[b3] Zannis V. I., Just P. W. & Breslow J. L. Human apolipoprotein E isoprotein subclasses are genetically determined. Am J Hum Genet 33, 11–24 (1981). 7468588 PMC1684875

[b4] Weisgraber K. H., Rall S. C. & Mahley R. W. Human E apoprotein heterogeneity. Cysteine-arginine interchanges in the amino acid sequence of the apo-E isoforms. J Biol Chem 256, 9077–9083 (1981). 7263700

[b5] Fullerton S. M. . Apolipoprotein E variation at the sequence haplotype level: implications for the origin and maintenance of a major human polymorphism. Am J Hum Genet. 67, 881–900 (2000). 10986041 10.1086/303070 PMC1287893

[b6] Strittmatter W. J. . Apolipoprotein E: high avidity binding to beta-amyloid and increased frequency of type 4 allele in late-onset familial Alzheimer’s disease. Proc. Natl. Acad. Sci. USA 90, 1977–81 (1993). 8446617 10.1073/pnas.90.5.1977 PMC46003

[b7] Saunders A. M. . Association of apolipoprotein E allele epsilon 4 with late-onset familial and sporadic Alzheimer’s disease. Neurology 43, 1467–72 (1993). 8350998 10.1212/wnl.43.8.1467

[b8] Corder E. H. . Gene dose of apolipoprotein E type 4 allele and the risk of Alzheimer’s disease in late onset families. Science 261, 921–923 (1993). 8346443 10.1126/science.8346443

[b9] Lambert J. C. . Meta-analysis of 74,046 individuals identifies 11 new susceptibility loci for Alzheimer’s disease. Nat Genet 45, 1452–8 (2013). 24162737 10.1038/ng.2802 PMC3896259

[b10] Farrer L. A. . Effects of age, sex, and ethnicity on the association between apolipoprotein E genotype and Alzheimer disease. A meta-analysis. APOE and Alzheimer Disease Meta Analysis Consortium. JAMA 278, 1349–56 (1997). 9343467

[b11] Ward A. . Prevalence of apolipoprotein E4 genotype and homozygotes (APOE e4/4) among patients diagnosed with Alzheimer’s disease: a systematic review and meta-analysis. Neuroepidemiology 38, 1–17 (2012). 22179327 10.1159/000334607

[b12] Gatz M. . Role of genes and environments for explaining Alzheimer disease. Arch Gen Psychiatry 63, 168–74 (2006). 16461860 10.1001/archpsyc.63.2.168

[b13] Genin E. . APOE and Alzheimer disease: a major gene with semi-dominant inheritance. Mol Psychiatry 16, 903–7 (2011). 21556001 10.1038/mp.2011.52 PMC3162068

[b14] Kim J., Basak J. M. & Holtzman D. M. The role of apolipoprotein E in Alzheimer’s disease. Neuron 63, 287–303 (2009). 19679070 10.1016/j.neuron.2009.06.026 PMC3044446

[b15] Xu Q., Bernardo A., Walker D., Kanegawa T., Mahley R. W. & Huang Y. Profile and regulation of apolipoprotein E (ApoE) expression in the CNS in mice with targeting of green fluorescent protein gene to the ApoE locus. J Neurosci 26, 4985–94 (2006). 16687490 10.1523/JNEUROSCI.5476-05.2006 PMC6674234

[b16] Grehan S., Tse E. & Taylor J. M. Two distal downstream enhancers direct expression of the human apolipoprotein E gene to astrocytes in the brain. J Neurosci 21, 812–22 (2001). 11157067 10.1523/JNEUROSCI.21-03-00812.2001 PMC6762321

[b17] Aoki K. . Increased expression of neuronal apolipoprotein E in human brain with cerebral infarction. Stroke 34, 875–80 (2003). 12649507 10.1161/01.STR.0000064320.73388.C6

[b18] Sun Y. . Glial fibrillary acidic protein-apolipoprotein E (apoE) transgenic mice: astrocyte-specific expression and differing biological effects of astrocyte-secreted apoE3 and apoE4 lipoproteins. J Neurosci 18, 3261–72 (1998). 9547235 10.1523/JNEUROSCI.18-09-03261.1998 PMC6792658

[b19] Smith J. D., Sikes J. & Levin J. A. Human apolipoprotein E allele-specific brain expressing transgenic mice. Neurobiol Aging 19, 407–13 (1998). 9880043 10.1016/s0197-4580(98)00076-1

[b20] Knouff C. . Apo E structure determines VLDL clearance and atherosclerosis risk in mice. J Clin Invest 103, 1579–86 (1999). 10359567 10.1172/JCI6172 PMC408371

[b21] Liraz O., Boehm-Cagan A. & Michaelson D. M. ApoE4 induces Ab42, tau, and neuronal pathology in the hippocampus of young targeted replacement apoE4 mice. Molecular Neurodegeneration 8:16 (2013). 23684315 10.1186/1750-1326-8-16 PMC3659080

[b22] Medway C. W. . *Apoe* variant p.V236E is associated with markedly reduced risk of Alzheimer’s disease. Mol Neurodegen 9, 11 (2014). 10.1186/1750-1326-9-11 PMC3995879 24607147

[b23] Yu C. E. . Epigenetic signature and enhancer activity of the human APOE gene. Hum Mol Genet 22, 5036–47 (2013). 23892237 10.1093/hmg/ddt354 PMC3836480

[b24] Chen H. P. . Screening reveals conserved and nonconserved transcriptional regulatory elements including an E3/E4 allele-dependent APOE coding region enhancer. Genomics 92, 292–300 (2008). 18718521 10.1016/j.ygeno.2008.07.009

[b25] Dynan W. S. Modularity in promoters and enhancers. Cell 58, 1–4 (1989). 2665940 10.1016/0092-8674(89)90393-0

[b26] Gotea V. . Homotypic clusters of transcription factor binding sites are a key component of human promoters and enhancers. Genome Res 20, 565–77 (2010). 20363979 10.1101/gr.104471.109 PMC2860159

[b27] Rao S. S. . A 3D map of the human genome at kilobase resolution reveals principles of chromatin looping. Cell 159, 1665–80 (2014). 25497547 10.1016/j.cell.2014.11.021 PMC5635824

[b28] Guenther C. A., Tasic B., Luo L., Bedell M. A. & Kingsley D. M. A molecular basis for classic blond hair color in Europeans. Nat Genet 46, 748–52 (2014). 24880339 10.1038/ng.2991 PMC4704868

[b29] Lettice L. A. . A long-range Shh enhancer regulates expression in the developing limb and fin and is associated with preaxial polydactyly. Hum Mol Genet 12, 1725–35 (2003). 12837695 10.1093/hmg/ddg180

[b30] Maas S. A. & Fallon J. F. Single base pair change in the long-range Sonic hedgehog limb-specific enhancer is a genetic basis for preaxial polydactyly. Dev Dyn 232, 345–8 (2005). 15637698 10.1002/dvdy.20254

[b31] Wang X. . A Polymorphic Antioxidant Response Element Links NRF2/sMAF Binding to Enhanced MAPT Expression and Reduced Risk of Parkinsonian Disorders. Cell Reports 15, 830–842 (2016). 10.1016/j.celrep.2016.03.068 PMC5063658 27149848

[b32] Soldner F. . Parkinson-associated risk variant in distal enhancer of α-synuclein modulates target gene expression. Nature 533, 95–9 (2016). 27096366 10.1038/nature17939 PMC5042324

[b33] Kelly D. P. & Scarpulla R. C. Transcriptional regulatory circuits controlling mitochondrial biogenesis and function. Genes Dev 18, 357–68 (2004). 15004004 10.1101/gad.1177604

[b34] Johar K., Priya A. & Wong-Riley M. T. Regulation of Na(+)/K(+)-ATPase by nuclear respiratory factor 1: implication in the tight coupling of neuronal activity, energy generation, and energy consumption. J Biol Chem 287, 40381–90 (2012). 23048038 10.1074/jbc.M112.414573 PMC3504753

[b35] Chang W. T., Chen H. I., Chiou R. J., Chen C. Y. & Huang A. M. A novel function of transcription factor alpha-Pal/NRF-1: increasing neurite outgrowth. Biochem Biophys Res Commun 334, 199–206 (2005). 15992771 10.1016/j.bbrc.2005.06.079

[b36] Choi Y. S., Lee K. U. & Pak Y. K. Regulation of mitochondrial transcription factor A expression by high glucose. Ann N Y Acad Sci 1011, 69–77 (2004). 15126285 10.1007/978-3-662-41088-2_8

[b37] Piantadosi C. A. & Suliman H. B. Mitochondrial transcription factor A induction by redox activation of nuclear respiratory factor 1. J Biol Chem 281, 324–33 (2006). 16230352 10.1074/jbc.M508805200

[b38] Tokusumi Y., Zhou S. & Takada S. Nuclear respiratory factor 1 plays an essential role in transcriptional initiation from the hepatitis B virus x gene promoter. J Virol 78, 10856–64 (2004). 15452206 10.1128/JVI.78.20.10856-10864.2004 PMC521811

[b39] Lezza A. M. . Increased expression of mitochondrial transcription factor A and nuclear respiratory factor-1 in skeletal muscle from aged human subjects. FEBS Lett 501, 74–8 (2001). 11457459 10.1016/s0014-5793(01)02628-x

[b40] Chandrasekaran S. & Bonchev D. Network Topology Analysis of Post-Mortem Brain Microarrays Identifies More Alzheimer’s Related Genes and MicroRNAs and Points to Novel Routes for Fighting with the Disease. PLoS One 11, e0144052 (2016). Erratum in: *PLoS One* **11**, e0151122 (2016). 26784894 10.1371/journal.pone.0144052 PMC4718516

[b41] Satoh J., Kawana N. & Yamamoto Y. Pathway Analysis of ChIP-Seq-Based NRF1 Target Genes Suggests a Logical Hypothesis of their Involvement in the Pathogenesis of Neurodegenerative Diseases. Gene Regul Syst Bio 7, 139–52 (2013). 10.4137/GRSB.S13204 PMC3825669 24250222

[b42] Braak H. & Braak E. Neuropathological stageing of Alzheimer-related changes. Acta Neuropathol. 82, 239–259 (1991). 1759558 10.1007/BF00308809

[b43] Mathelier A. . JASPAR 2014: an extensively expanded and updated open-access database of transcription factor binding profiles. Nucleic Acids Res 42, D142–D147 (2014). 24194598 10.1093/nar/gkt997 PMC3965086

[b44] van den Hoven R. . Putative regulation mechanism for the MSTN gene by a CpG island generated by the SINE marker Ins227bp. BMC Vet Res 11, 138 (2015). 26100061 10.1186/s12917-015-0428-3 PMC4476204

[b45] Benítez-Bellón E., Moreno-Hagelsieb G. & Collado-Vides J. Evaluation of thresholds for the detection of binding sites for regulatory proteins in Escherichia coli K12 DNA. Genome Biol 3, RESEARCH0013 (2002). 10.1186/gb-2002-3-3-research0013 PMC88811 11897025

[b46] Schneider D. T. & Stephens R. M. Sequence logos: a new way to display consensus sequences. Nucleic Acids Research 18, 6097–6100 (1990). 2172928 10.1093/nar/18.20.6097 PMC332411

